# Inflammatory myofibroblastic bladder tumor: A very rare presentation

**DOI:** 10.1016/j.eucr.2021.101863

**Published:** 2021-09-27

**Authors:** Raphael Flavio Fachini Cipriani, Alexandre Cavalheiro Cavalli, Joaquim Lorenzetti Andrade, Luciano Ricardo Sfredo, Ivam Vargas Martins da Silva, Ingridy de Souza Digner

**Affiliations:** a Urology Resident, Universidade Federal do Paraná, Hospital de Clínicas, Curitiba, Brazil; b Division of Urology, Universidade Federal do Paraná, Hospital de Clínicas, Curitiba, Brazil; c Urology Internship, Universidade Federal do Paraná, Hospital de Clínicas, Curitiba, Brazil

**Keywords:** Urinary bladder neoplasms, Hematuria, Cystectomy

## Abstract

Inflammatory myofibroblastic tumor (IMT) is a rare benign lesion with similarities to malignant lesions due to possible aggressive behavior. Although highly uncommon, this condition usually occurs in lungs and retroperitoneum. The involvement of the genitourinary tract represents a singular occasion. May present with nonspecific manifestations such as painless hematuria, dysuria, voiding urgency and low abdominal pain. We describe a Case of a 55-year-old patient who presented to the urology service complaining of hematuria. Imaging studies showed a 62mm lesion on the upper right side of the bladder and the diagnosis of IMT was confirmed by immunohistochemical evaluation after laparoscopic partial cystectomy.

## 1 Introduction

Inflammatory myofibroblastic tumor (IMT) is a rare benign lesion that has similarities to malignant lesions due to possible aggressive behavior, although its specific potential for malignancy has not been determined.^1^ It is a soft tissue tumor, which evolves through myofibroblastic differentiation and is characterized by the infiltration of inflammatory cells into the tissue. The etiology of its development remains uncertain, but there are studies that suggest possible occurrence secondary to infections, trauma or pelvic surgeries.^2^^,^^3^

## 2 Case presentation

**Fig. 1:**
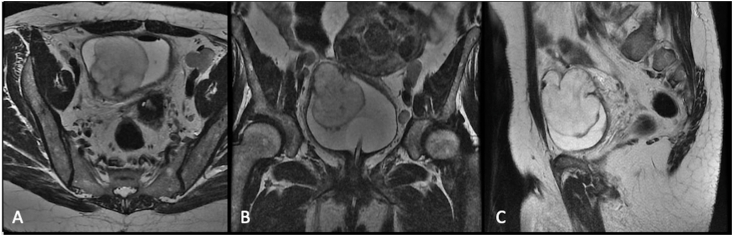
MRI of the Pelvis in the T2 phase in 3 incidences: A) Axial, B) Coronal and C) Sagittal – evidencing injury to the right posterior face of the bladder surpassing the muscular layer and serous of the bladder with intraperitoneal invasion of about 23 mm. Lesion measurements 62 × 53 × 54 mm. Contact of the upper face of the lesion was noted for about half of the adjacent parts of the round ligament.

**Fig. 2:**
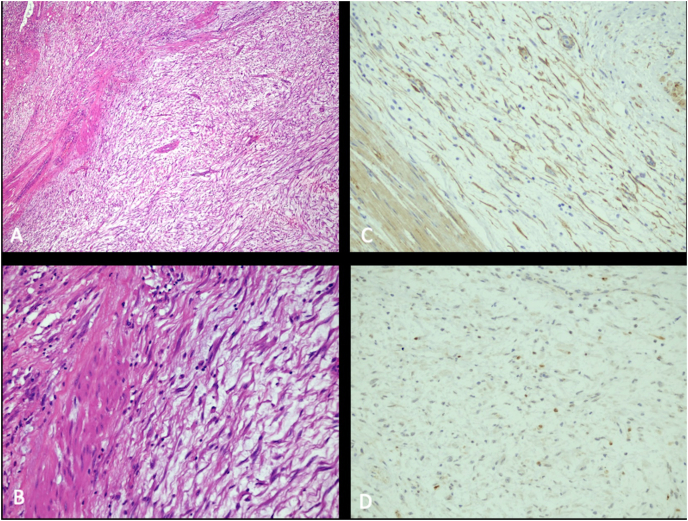
Fragments of bladder wall in hematoxylin-eosin on the left column (A and B), showing spindle-cell proliferation without atypical in lamina propria, abundant stroma of myxoid pattern, minimal pleomorphism and absence of necrosis; Immunohistochemical analysis of bladder lesion on the right column, that exhibits muscle markers (A), actin and desmine, and low proliferative index (B), Ki67 less than 10%, which is compatible with IMT.

AC, 55 years old, woman, presented to the Urology Service complaining of hematuria. His previous medical history includes indolent lymphoma and hypothyroidism. During cystoscopy, a massive lesion was identified in the bladder fundus, right lateral wall, occupying 2/3 of the bladder lumen, with area of necrosis and 4cm pedicled component of length, with 8cm of average, it was confirmed with a Magnetic Resonance Imaging (MRI) of the pelvis (Fig. 1). Pathological study demonstrated spindle-cell proliferation without atypical in lamina propria, with abundant myxoid stroma (Fig. 2).

**Fig. 3:**
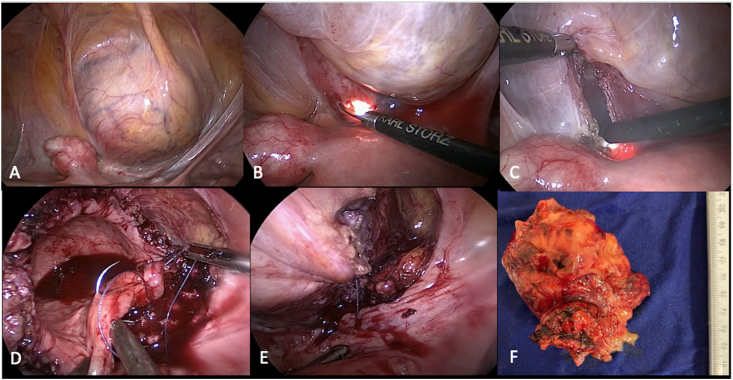
Laparoscopic partial cystectomy: A) Tumor mass bulging the bladder fundus; B) Section of the posterior wall of the bladder guided by cystoscopy; C) Lateral cystotomy and delimitation of the lesion; D) Bladder suture; E) Final aspect; F) Surgical specimen containing partial cystectomy product.

The patient had received blood transfusion at admission and after two weeks, when the serum hemoglobin levels were 4.9 g/dL, and in the preoperative period, when the levels were 7.3 g/dL. She underwent partial laparoscopic cystectomy with a combined approach (Fig. 3). Post-op evolution was good, with hospital discharge in the fifth day. Immunohistochemical analysis revealed positivity for smooth muscle actin (1A4), CK AE1/AE3, ALK, Desmina and Ki67, findings compatible with the diagnosis of inflammatory myofibroblastic tumor (Fig. 1).

## 3 Discussion

Just over 100 cases of bladder IMT have been reported worldwide since 1980, when the syndrome was first described.^1^ Thus, it is an uncommon lesion and without sufficient evidence in the literature that allows consensus in the diagnostic and therapeutic approach. When present, this condition usually occurs in the lungs and retroperitoneum, and tends to occur in younger populations. The involvement of the genitourinary tract represents an even more singular occasion, being apparently more prevalent, in this Case, in men over 40 years of age. Epidemiological characteristics, however, do not allow predictability due to the reduced number of cases.^1^

The IMT is a soft tissue tumor, which evolves through myofibroblastic differentiation. Its etiology development remains uncertain, but studies suggest possible occurrence secondary to infections, trauma or pelvic surgeries. When occurs in the genitourinary tract, IMT most often affects the bladder. In this Case, may present nonspecific manifestations, such as dysuria, voiding urgency and low abdominal pain, besides presenting hematuria as the most common manifestation.^2^^,^^4^ Thus, the case fits the predicted period of onset of symptoms and presents nonspecific evidence like the cases already reported.

For the diagnostic approach, imaging study shows an infiltrating mass, but does not allow the differential diagnosis of the lesion, due to the similarities with malignant neoplasms. Thus, performance of these tests will be important to suggest suspicious lesions, but it becomes secondary in diagnostic confirmation, which depends on biopsy and histological analysis. Microscopic examination will reveal presence of myofibroblastic fusiform cells associated with inflammatory components.^2^^,^^3^ For treatment, surgical resection remains the best conduct, with the possibility of an open, laparoscopic or transurethral approach. Although transurethral resection of the tumor is more commonly performed, partial laparoscopic cystectomy is related to a lower recurrence rate.^5^

## 4 Conclusion

IMT is a benign lesion that has similarities with invasive tumors. Because it is a rare condition, there are no defined protocols for the diagnostic and therapeutic approach. Thus, Case reports are a relevant way to contribute to the advancement of knowledge about such a condition. This report highlights the importance of adequate identification of the pathology to avoid misguided interventions.

## Funding

This research did not receive any specific grant from funding agencies in the public, commercial, or not-for-profit sectors.
